# Suppressive Effect of Constructed shRNAs against Apollon Induces Apoptosis and Growth Inhibition in the HeLa Cell Line

**DOI:** 10.7508/ibj.2016.03.003

**Published:** 2016-07

**Authors:** Saeideh Milani, Mojgan Bandehpour, Zohreh Sharifi, Bahram Kazemi

**Affiliations:** 1 Department of Biotechnology, School of Medicine, Shahid Beheshti University of Medical Sciences, Ewin, Chamran highway, Tehran, Iran;; 2 Department of Biotechnology, School of Advanced Technologies in Medicine, Shahid Beheshti University of Medical Sciences, Tehran, Iran;; 3 Blood Transfusion Research Center, High Institute for Research and Education in Transfusion Medicine, Hemmat EXP., Tehran, Iran;; 4 Cellular and Molecular Biology Research Center, Shahid Beheshti University of Medical Sciences, Tehran, Iran

**Keywords:** Coronary artery disease, Single nucleotide polymorphisms, Genetic association study, Iran

## Abstract

**Background:**

Cervical cancer is the second most common female cancer worldwide. Inhibitors of apoptosis proteins (IAPs) block apoptosis; therefore, therapeutic strategies targeting IAPs have attracted the interest of researchers in recent years. Apollon, a member of IAPs, inhibits apoptosis and cell death. RNA interference is a pathway in which small interfering RNA (siRNA) or shRNA (short hairpin RNA) inactivates the expression of target genes. The purpose of this study was to determine the effect of constructed shRNAs on apoptosis and growth inhibition through the suppression of apollon mRNA in HeLa cell line.

**Methods:**

Three shRNAs with binding ability to three different target sites of the first region of apollon gene were designed and cloned in pRNAin-H1.2/Neo vector. shRNA plasmids were then transfected in HeLa cells using electroporation. Down-regulation effects of apollon and the viability of HeLa cells were analyzed by RT-PCR, lactate dehydrogenase assay**, **and MTT assay, respectively. Also, the induction and morphological markers of apoptosis were evaluated by caspase assay and immunocytochemistry method.

**Results:**

The expression of shRNA in HeLa cells caused a significant decrease in the level of apollon mRNA1. In addition, shRNA1 effectively increased the mRNA level of Smac (as the antagonist of apollon), reduced the viability of HeLa cells and exhibited immunocytochemical apoptotic markers in this cell line.

**Conclusion:**

Apollon gene silencing can induce apoptosis and growth impairment in HeLa cells. In this regard, apollon can be considered a candidate therapeutic target in HeLa cells as a positive human papillomavirus cancer cell line.

## INTRODUCTION

It has been well documented that human papillomavirus (HPV) has a key role in cervical cancer, which is the second most prevalent cancer among women worldwide and the most frequent in developing countries^[^^1^^,^^2^^]^. The inhibitors of apoptosis proteins (IAPs) are a family of proteins involved in a variety of cell signaling pathways such as anti-apoptotic process^[^^3^^]^. These proteins are prominently expressed in transformed cell lines as well as in many human tumors^[^^4^^]^. The inhibition of some IAPs, including the X-linked inhibitor of apoptosis protein and survivin has been reported to have therapeutic potential or a prognostic value for cervical cancers. Espinosa *et al*.^[^^5^^]^ have found that the X-linked inhibitor of apoptosis protein is responsible for the relapse of cervical cancer. Also, they showed that survivin is up-regulated in these patients. 

Apollon (BRUCE or BIRC6), which is a 528-kDa membrane-associated IAP, involves in cell survival and can promote ubiquitination of the IAP antagonist Smac, a group of cysteine proteases that have a central executive role in apoptosis by binding to apollon. It contains one baculoviral IAP repeat domain at its N-terminal region and a C-terminal E2 motif that can form thioester bonds with ubiquitin^[^^6^^]^. 

According to Dong *et al.*^[^^7^^]^, the overexpression of apollon is related to malignant progression and poor response to treatment in lung cancer. Low and his collogues^[^^8^^]^ have shown that the elevated expression of apollon protein has a significant role in prostate cancer progression and treatment resistance. This result has been also indicated in different studies carried out in cells from gliomas^[^^9^^]^, lung cancers^[^^10^^]^, fibro-sarcomas^[^^11^^]^, osteosarcomas^[^^12^^]^, breast cancers, and colon cancers^[^^13^^]^. Thus, drugs or treatment strategies, which have potential for inhibiting apollon as well as restoring the apoptotic signaling, can be used for the elimination of cancer cells^[^^14^^]^.

One of the powerful tools for down-regulation of IAP genes in cancerous cells is RNA interference (RNAi). It can knockdown the gene expression in mammalian cancerous cells^[^^15^^,^^16^^]^. On the other hand, the presentation of short hairpin RNA (shRNA) under H1 promoter ensures that the shRNA is always expressed and can be cleaved by the cellular machinery into siRNA. In the present study, the shRNA expression vector (pRNA-H1.2/Neo) was used due to some of its advantages to siRNA. One of the most benefits of using shRNA is continuous expression by the host cell, which results in its more long-lasting or long-term effects in transfected cells^[^^17^^]^. The purpose of the present study was to estimate the effects of apollon knockdown on the proliferative potential and apoptosis induction in the HeLa cell line using RNAi.

## MATERIALS AND METHODS

**Construction of shRNA plasmids**

Three shRNA fragments from the first coding region of human apollon gene (NM_016252.3) were selected. The HPV-shRNAs were cloned into the *Bam*HI and *Hind*III cloning sites of the pRNAin-H1.2 Neo expression vector (Table 1). The accuracy of the constructed plasmids was confirmed by restriction mapping using *Hind*III and *Bam*HI enzymes (Fermentas, USA) and sequencing.

**Cell culture and transfection**

Human cervical carcinoma cell line (HeLa cell line) was obtained from Blood Transfusion Research Center (Tehran, Iran). The cells were grown in DMEM (Gibco, Germany) supplemented with 10% fetal calf serum (Gibco, Germany), 100 units/ml penicillin (Sigma, USA), and 100 µg/ml streptomycin (Sigma, USA) in a humidified chamber under 5% CO_2_ at 37ºC. The cells were then transfected with 5 μg shRNA plasmid using a multiporator (Eppendorf, Germany). Twenty hours after the transfection, 3 µl G418 (10 mg/ml), as a selection marker, was used to choose the stable transfected HeLa cell line. The experimental subjects were four groups consisting of three interference groups (shRNA-transfected cells) and mock group transfected with pRNAin-H1.2 Neo vector. To determine the transfection efficacy, the pEGFP-N1 plasmid (Clontech, UK) was utilized.

**Quantitative RT-PCR**

After 48-h incubation, the expression levels of apollon and Smac in transfected cells were analyzed by RT-PCR using specific primers. Total RNA was extracted by the Total RNA Purification Kit (Gena Bioscience, Germany) according to the manufacturer’s instruction. The complementary DNA was synthesized by *Moloney Murine Leukemia Virus Reverse Transcriptase* (Fermentas, Lithuania) at 42ºC for 1 hour. RT-PCR was performed with 10 μl Accupower**®** 2× Greenstar qPCR Master Mix (Bioneer, Korea), 1 μg cDNA and 4 pmol each of the specific primers using Rotor Gene 6000 (Corbett Research, Germany) in a total volume of 20 μl. The thermal cycling conditions were carried out in an initial denaturation steps at 94ºC for 5 min, followed by 45 cycles of 94ºC for 5 s, 50ºC for 8 s, and 72ºC for 10 s. Amplification of β-actin, as the housekeeping gene, was also carried out. The primers were as below:

Apollon: 5’-AGTGCAACGATGTGCCAT-3’/5’-GCT AACCAACAGAGAGTA-3’

Smac/Diablo: 5’-ATCATAGGAGCCAGAGCTG-3’/ 5’-GCCAGTTTGATATGCAGCT-3’

β-actin: 5’-GATGAGTATGCCTGCCGTGTG-3’/5’-C AATCCAAATGCGGCATCT-3’ 

**Table 1:** shRNA sequence against apollon mRNA

**Name**	**Sequences (5** **′-** **3** **′** **)**
shRNA # 1	5′CACCCTGCGCTCAACGCCATCTTCAAGAGAGATGGCGTTGAGCGCAGGGTG3′
shRNA # 2	5′CATGCTGGAATGTTGACGTTATTCAAGAGATAACGTCAACATTCCAGCATG3′
shRNA # 3	5′TGGGAGATTGTTGCAAATGTTCAAGAGACATTTGCAAGAATCTCCCAC3′

**MTT assay**

To evaluate the proliferation of shRNA-transfected cells, after the incubation period, 100 μl MTT (5 mg/ml) was added to each well and then incubated at 37ºC for 2 hours. Then 100 µl DMSO (Sigma, USA) was added to solubilize the formazan crystals. The absorbance of the samples was calculated using an ELISA plate reader (Tecan, Sweden) in a wavelength of 490 nm, and the reference wavelength was considered at 690 nm. 

**Lactate dehydrogenase (LDH) assay**

To measure the viability and cytotoxicity of HeLa cells transfected with shRNAs, LDH activity was measured by a LDH cytotoxicity assay kit II (Abcam, UK) according to the manufacturer’s instructions.

**Immunocytochemistry** **assay**

For detection of immunocytochemical apoptotic markers, cells were cultured on gelatin-coated coverslips. HeLa cells were fixed with 4% paraformaldehyde, rinsed twice with PBS and permeabilized with 0.3% Triton X-100. The cells were incubated in 2% BSA at room temperature for 1 hour, followed by incubation with the primary anti-apollon antibody (1/500) (A1592-Abcam, UK). Then the cells were incubated with FITC-conjugated secondary antibody (Bioorbyt, UK). Nuclei were counter stained with DAPI, and the images of the stained cells were taken using an immunofluorescence microscope (Ziess, Germany). The apoptosis was studied on the base of characteristic changes in nuclear morphology. 

**Caspase assay**

Caspase-9 activity was assayed by the Colorimetric Caspase-9 Assay Kit (Abcam, UK) according to the manufacturer’s protocol. 

**Statistical analysis**

All statistical analyses were performed using SPSS 16. Each experiment was carried out in triplicate for all data (n=3). Data were expressed as mean±standard error of the mean. Differences between the control and shRNA-transfected cells in terms of growth and viability of the cells were analyzed using one-way analysis of variance (ANOVA) and the independent samples *t*-test. The results were regarded statistically significant at *P*<0.05. Also, an average expression value (*E *value) indicating gene regulation was calculated using REST software, and 95% confidence intervals was used for expression ratios.

## RESULTS

**shRNA plasmid construction**

In the current study, shRNA expression vectors were as constructed against the apollon gene under the control of H1 promoter. Different studies have demonstrated that shRNA effects depend on the target sites^[^^18^^-^^20^^]^. Therefore, we chose three different target sites and constructed three vectors, including shRNA1, 2, and 3 plasmids (Table 1).

**Quantitative real-time PCR**

**Fig. 1:**
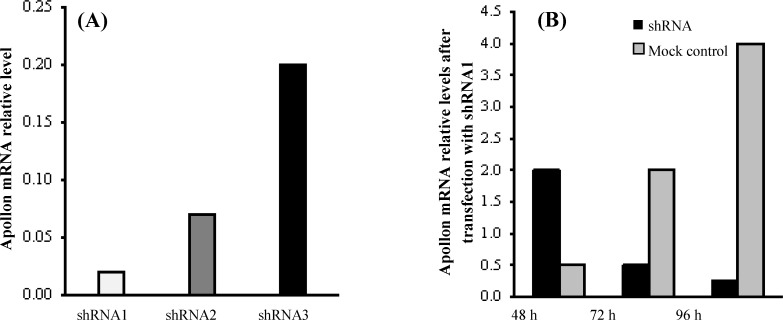
Relative RNA expression of apollon gene in HeLa cells transfected with different shRNAs compared to mock control cells (A). Comparison of apollon mRNA expression in HeLa cells at 48, 72, and 96 h after shRNA1 transfection is shown in the Figure (B). The mRNA expression of apollon was normalized with β-actin. An average expression value (*E* value) indicating gene regulation was calculated using REST software. Also, 95% confidence intervals were used for expression ratios

**Fig. 2:**
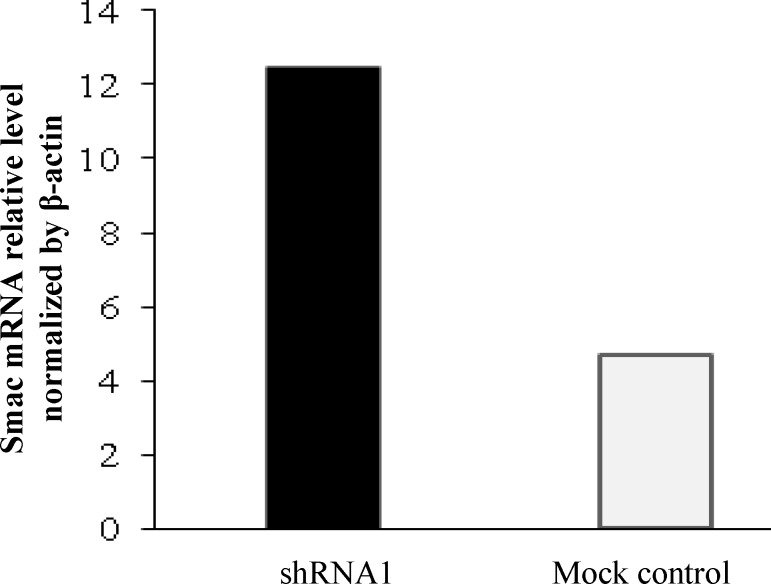
Up-regulation of Smac after apollon knockdown shown 48 h after the transfection of the HeLa cells with shRNA1 plasmid. The mRNA expression of Smac was normalized with β-actin. An average expression value (*E* value) indicating gene regulation was calculated using REST software, and 95% confidence intervals were used for expression ratios

The results from real-time quantitative RT-PCR indicated that all three designed shRNAs can decrease the mRNA level of apollon (shRNA1, 2, and 3; 98%, 93% and 80%, respectively). Analysis of the RT-PCR results using REST software showed that apollon was down-regulated in the sample group (in comparison to the control group) by a mean factor of 0.02, 0.07, and 0.2 after transfection of the HeLa cells with shRNA1, 2, and 3, respectively (Fig. 1). However, we selected the most efficient shRNA1 for the further studies. The inhibition extent was increased over time and reached its maximum at 96 h (48, 72, and 96 h; 10.8%, 43.7%, and 87.5%, respectively) (Fig. 1). Additionally, the analysis of RT-PCR using REST software showed that the expression of Smac mRNA, as an antagonist of apollon in HeLa cells transfected with the apollon shRNA1, was increased significantly by a mean factor of 12.4 (2.5-folds) compared to the mock control group (Fig. 2).

**Cell viability**

**Fig. 3:**
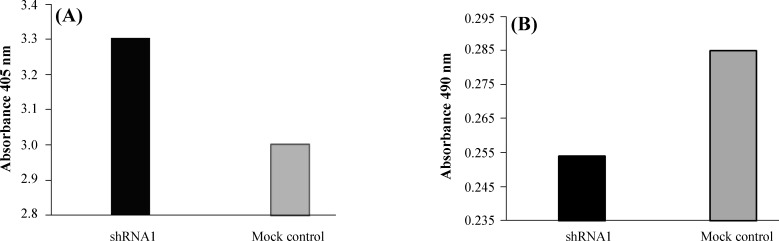
Effect of apollon down-regulation on viability of the HeLa cells. Cell viability was measured using MTT and LDH assays. (A) and (B) show LDH and MTT assays, respectively. Each bar represents the mean value±standard deviation (SD) of triplicate.* P*<0.05 was compared to the control cell group

Cell viability was assessed by two methods and assessed by MTT assay at a 48-h interval. There was a difference in the cell viability between shRNA1 plasmid and non-transfected control cells, with a significant reduction in the growth of the HeLa cell lines following the expression of Apollon-specific shRNA. LDH was considered as the second cell viability parameter. It is a stable enzyme that presents in all cell types and suddenly is released into the cell culture medium upon the damage of the plasma membrane. As it was anticipated, the viability of HeLa cells transfected with shRNA1 plasmid was significantly different from the control cells (Fig. 3).

**Immunocytochemistry assay**

**Fig. 4:**
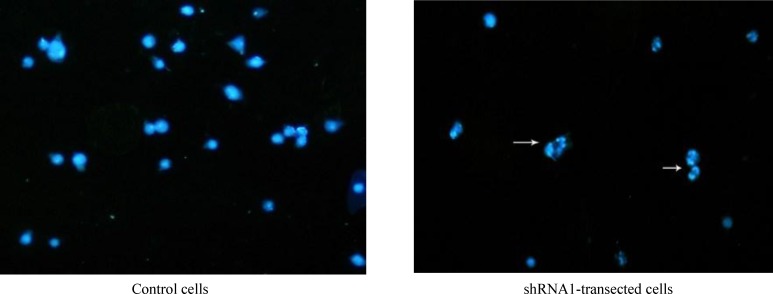
Effect of apollon gene silencing on the induction of apoptosis. Immunocytochemical staining using anti-apollon in HeLa cells. DAPI staining was employed as a nuclear counter stain. Arrows show apoptosis induction in cells

On the basis of immunocytochemistry results, the apoptosis process was detected in the transfected HeLa cells. Characteristic changes in nuclear morphology, including chromatin condensation and nuclear fragmentation were demonstrated using immune-fluorescence microscopy. As shown in Figure 4, down-regulation of apollon induced apoptosis phenotype in transfected cells. Also, caspase catalytic activity was further assessed in cell lines 48 h after the transfection. The results revealed that in HeLa cells, apollon knockdown induced 1.2-fold increasing in caspase-9 catalytic activity compared with control cells.

## DISCUSSION

HPVs account for 5% of all cancers globally, which resemble the load caused by both hepatitis B and *Helicobacter pylori*. Moreover, a high percentage of cervical cancer is caused by HPV infections all over the world^[^^21^^,^^22^^]^. 

In the present study, we selected the cervical cancer HeLa cell to evaluate the effects of RNAi-mediated apollon gene silencing. Different studies have demonstrated shRNA effects on the target sites^[^^18^^-^^20^^]^. In this regard, we chose three different target sites for the apollon gene and constructed three vectors, shRNA1, 2, 3 plasmids. The apollon gene comprises 75 exons, which result in a transcript of 16,066 bp^[^^23^^]^. To inhibit the transcription at primary steps and to prevent synthesis of incomplete apollon mRNA, which may have undesirable impact on our results, we selected those exons that almost belonged to the beginning region of apollon mRNA (shRNA1: exon 1, shRNA2: exon 14, and shRNA3: exon 9).

RNAi is a molecular tool with potential in therapy of diverse human diseases, especially cancers^[^^24^^,^^25^^]^. There is a relationship between IAP overexpression and unfavorable clinical features^[^^26^^]^. Therefore, IAPs targeting can induce apoptosis in malignant cells^[^^27^^,^^28^^]^. Chen *et al.*^[^^9^^]^ deduced that apollon guards SNB-78 cells from undergoing apoptosis and plays a role in tumorigenesis and drug resistance of this cell line. Apollon knockout in mice results in embryonic or neonatal lethality^[29]^. In one study, Espinosa *et al.*^[^^5^^]^ explained that the down-regulation of apollon has an important role in inhibition of human cervical cancer cell proliferation. Apollon ubiquitinates and facilitates proteasomal degradation of mature Smac and pro-caspase-9^[^^15^^]^ and inhibits its cleavage to the mature form^[^^30^^]^. It has been shown that the promotion of the apoptosis induced by certain apoptotic stimuli can be a result of Smac precursor overexpression^[^^25^^]^. Ren *et al.*^[^^31^^] ^demonstrated that the inactivation of Bruce, the murine homologue of apollon, by deletion of the C-terminal UBC domain induces a strong apoptotic response. Qiu *et al.*^[^^32^^]^ reported that in response to certain apoptotic stimuli (e.g. etoposide), ubiquitination and proteasomal degradation of apollon are promoted by Nrdp1. 

In the current investigation, real-time quantitative RT-PCR results revealed that all three designed shRNAs can decrease the mRNA level of apollon; however, we selected the most efficient one (shRNA1) for the further study. Additionally, the expression of Smac mRNA, as an antagonist of apollon in HeLa cells transfected with the apollon shRNA, was increased significantly compared to mock control based on real-time PCR.

Significant growth inhibition and cell death have been shown in apollon-shRNA1 transfected HeLa cell line through MTT and LDH assays. Immuno-cytochemistry test also indicated the morphological patterns of apoptosis in the nucleus, including chromatin condensation and nuclear fragmentation. Furthermore, a decrease in endogenous apollon led to an apparent increase in the levels of active caspase-9, which confirms that apollon knockdown activates a caspase-dependent apoptotic process.

In conclusion, these results propose that targeting the apollon by shRNA plasmids can significantly decrease apoptotic resistance in a model cancerous cell line, HeLa. However, results from *in vivo* studies will be required to generalize our results as a promising method for cervical cancer therapy.
